# Prevalence and Risk Indicators for Anal Incontinence among Pregnant Women

**DOI:** 10.1155/2013/947572

**Published:** 2013-05-29

**Authors:** Katariina Laine, Finn Egil Skjeldestad, Leiv Sandvik, Anne Cathrine Staff

**Affiliations:** ^1^Department of Obstetrics and Department of Gynaecology, Oslo University Hospital, Ullevål, Pb 4965, Nydalen, 0424 Oslo, Norway; ^2^Faculty of Medicine, University of Oslo, Norway; ^3^Women's Health and Perinatology Research Group, Department of Clinical Medicine, University of Tromsø, 9019 Tromsø, Norway; ^4^Unit of Biostatistics and Epidemiology, Oslo University Hospital, Pb 4965, Nydalen, 0424 Oslo, Norway

## Abstract

The aim of this study was to assess the prevalence and risk factors of anal incontinence in an unselected pregnant population at second trimester. A survey of pregnant women attending a routine ultrasound examination was conducted in a university hospital in Oslo, Norway. A questionnaire consisting of 105 items concerning anal incontinence (including St. Mark's score), urinary incontinence, medication use, and comorbidity was posted to women when invited to the ultrasound examination. *Results*. Prevalence of self-reported anal incontinence (St. Mark's score ≥ 3) was the lowest in the group of women with a previous cesarean section only (6.4%) and the highest among women with a previous delivery complicated by obstetric anal sphincter injury (24.4%). Among nulliparous women the prevalence of anal incontinence was 7.7% and was associated to low educational level and comorbidity. Prevalence of anal incontinence increased with increasing parity. Urinary incontinence was associated with anal incontinence in all parity groups. *Conclusions*. Anal incontinence was most frequent among women with a history of obstetric anal sphincter injury. Other obstetrical events had a minor effect on prevalence of anal incontinence among parous women. Prevention of obstetrical sphincter injury is likely the most important factor for reducing bothersome anal incontinence among fertile women.

## 1. Introduction

Anal incontinence is a bothersome ailment associated with many health complaints and discomfort in daily life: hygienic problems, limitations in occupational and social life, sexual dysfunction, reduced quality of life, and altered self-esteem. Anal incontinence (AI) is defined as involuntary loss of flatus or feces [1]. Prevalence and severity of anal incontinence are measured by patient self-reporting and no objective assessment methods exist.

Obstetric anal sphincter injury (OASIS) is one of the main causes for female AI reported in nonpregnant women. Additionally, multiple vaginal deliveries can increase the risk of AI regardless of anal sphincter injury [2, 3]. Age, obesity and medical conditions such as diabetic neuropathy and gastrointestinal disorders also increase the risk of anal incontinence [2, 4, 5].

Prevalence of anal incontinence among women differs largely (2–28%) in previous studies and differs between different study populations [4–6]. Postpartum studies show a high prevalence of AI in women having suffered from OASIS, 38–59% [6–8]. Women attending gynecological outpatient clinics have higher prevalence of AI (16–28%) compared with the general female population (4.4%) [2, 5]. Women with pelvic floor disorders have higher prevalence of AI than women without pelvic floor disorders. Community-based studies show differences in prevalence of AI between age groups, with increasing prevalence by increasing age [4, 5, 9]. Most frequent AI is found among nursing home residents (50–60%), among the oldest women with frequent additional complaints and comorbidity [10].

Few previous studies have assessed the prevalence of anal incontinence in a female population of fertile age, and few studies have included nulliparous women [11–14]. 

The aim of this study was therefore to assess the prevalence and risk factors for anal incontinence in an unselected female population across parity groups in second trimester of pregnancy.

## 2. Material and Methods

This study is part of a comprehensive perineum research study, which was approved by the Regional Committee for Medical Research Ethics in South-Eastern Norway (ref. S-08810d/20941) and the Institutional Personal Data Officer.

This study was conducted as a survey of pregnant women attending free of charge routine ultrasound examination at second trimester, from September 2009 to August 2010, in a large university hospital in Oslo, Norway. The pregnant women attending the ultrasound screening in our hospital represent a nonselected population from the entire Oslo area. All pregnant women in Norway are offered a free of charge second trimester routine ultrasound examination in gestational week 18–20, and 98% attend. In our hospital, this routine ultrasound is performed by specially educated midwives at the fetal medicine unit. The hospital receives admission notes from the local general practitioners when the woman is pregnant in the first trimester. The invitation to participate in our study, the questionnaire, and the informed consent were included as a part of the invitation to the routine ultrasound appointment. Midwives performing the routine ultrasound examination reminded the women of the study and collected the questionnaire and the signed informed consent. From the 7256 women who were posted a questionnaire, 973 women were not found in our postpartum labor ward database: they did not achieve 18 weeks pregnancy (pregnancy loss), they did not deliver in our hospital, or they had moved out of Oslo area or Norway, resulting in 6283 women eligible for study participation. We received 2851 filled-out questionnaires from the participants. Four women returned two questionnaires (twice during the same pregnancy), and one woman returned the questionnaire shortly after the index delivery. Thus, five filled-out forms were excluded from the analyses and the study group consisted of 2846 (of 6283 invited) women, resulting in a response rate of 45%.

The questionnaire consisted of 105 questions concerning anal and urinary incontinence, general health condition, drug use, and worries concerning pregnancy and delivery. The major part of the questions was chosen from validated questionnaires such as Due and Ottesen [15], St. Mark's [16], NUGG [17], HUNT [18], and Cambridge worry scale (CWS) [19, 20]. Additionally, we collected demographic data, obstetrical history, educational level, household income, and country of origin of the participant.

Anal incontinence was identified by self-reported leakage of gas, loose, or solid stools, lack of ability to defer defecation for 15 minutes (fecal urgency), use of pads or plugs, and alteration of lifestyle described in St. Mark's score. We defined anal incontinence as a St. Mark's score 3 or above (of maximal score 24). Women with St. Mark's score from 0 to 2 were analyzed as a control group (no or infrequent AI). Fecal incontinence was defined as self-reported leakage of loose or solid tools. Urinary incontinence was defined as self-reported symptoms of stress or urge urinary incontinence.

Parity was adjusted to history of cesarean delivery. Thus, women with cesarean delivery only (never having delivered vaginally before) were categorized as “vaginal primiparous.”

The data were analyzed by using PASW (Predictive Analytics SoftWare, SPSS Inc., version 19.0, Chicago, IL, USA). Continuous data were categorized and the independent variables are presented as frequencies. Univariate analysis was performed to identify significant risk factors for anal incontinence. Univariate analyses were performed by Chi-square test. A significance level of 5% was chosen in all analyses. Variables with *P* ≤ 0.05 were included in a multivariate logistic regression analysis. The results from this regression analysis are presented as adjusted odds ratios (aORs) for AI with 95% CI. For each model the assumptions underlying a valid logistic regression analysis were checked and found to be adequately met.

## 3. Results

Prevalence of self-reported anal incontinence (defined as St. Mark's score 3 or more) in the entire study group was 8.4% (238/2846). Most of the women (80.3%) reported complete anal continence (2268/2846) with St. Mark's score 0, and 11.3% (322/2846) women reported infrequent AI with St. Mark's score 1 or 2. Inability to control flatus was the most frequent complaint, reported by 18.0% (513/2846). Of these, 385 women reported episodes of flatus incontinence without fecal incontinence. Fecal incontinence was reported by 6.0% (171/2846) and fecal urgency by 3.2% (90/2846) of the women. 

Urinary incontinence was reported by 19% (547/2846) of the women. Urinary incontinence (UI) was significantly associated with reported anal incontinence among all parity groups and 32.4% of the women with AI also reported UI (*P* < 0.001). Prevalence of UI was threefold among parous women compared to nulliparous women and increased slightly with increasing vaginal parity (*P* < 0.001) (data not shown).

The majority of the 2846 women had answered the questionnaire when they were in the second trimester (84%), 12.2% in the first trimester, and 1.2% in the third trimester. 

Most of the participating women were nulliparous (63%) (1792/2846). The majority of the participating women were Norwegian (77.5%). Non-Western origin was reported by 13.5% of the women. Mean age of the participating women was 31 years. Mean height was 168 cm and mean weight was 66.7 kg. Mean BMI was 23.6, range 16.0–42.4. Smoking was infrequent; only 2% (57/2851) women reported that they smoked during this pregnancy (Table 1).

There was a significant difference in prevalence of AI among women with different obstetric histories. Prevalence of AI (defined as St. Mark's score of 3 or above) increased with increasing vaginal parity (data not shown).

### 3.1. Nulliparous Women

Of the nulliparous women, 7.8% (139/1792) reported anal incontinence. In the univariate analysis, non-Western background, low household income, being unmarried or single, low educational level, age 35 or more, answering the questionnaire in the first trimester (as opposed to second trimester), dermatological disease, ulcerative stomach disease, hypertension, rheumatoid arthritis, and muscle-skeletal complaints were significantly associated with anal incontinence among the nulliparous women (Tables 1 and 2).

In the multivariate analysis, low educational level, dermatological disease, and rheumatoid arthritis remained as significant factors for AI (Table 2).

### 3.2. Parous Women

After excluding the 140 women with previous cesarean delivery only, the subgroup of vaginal parous women consisted of 914 women. Overall anal incontinence among parous women was 9.8% (90/914). In the group of women with one previous vaginal delivery, 8.5% (61/714) reported AI, whereas the group of women with more than one previous vaginal delivery, as many as 14.5% (29/200), reported AI (*P* = 0.004).

Of the parous women, 15.9% had previously delivered at least one macrosomic (>4000 g) infant (145/914), 156 reported previous delivery with vacuum extraction, and 24 women reported a previous forceps delivery. An obstetric history with instrumental delivery or a macrosomic infant was not associated with AI. Previous delivery with OASIS was reported by 41 women (4.5%) and was strongly associated with AI. 

In the univariate analysis, previous delivery with OASIS, non-Western background, low household income, being unmarried or single, low educational level, age 35 or more, BMI 25 or more, dermatological disease, and use of pain killers were significantly associated with anal incontinence among the parous women (Tables 1 and 2).

In the multivariate analysis, previous delivery complicated with OASIS and dermatological disease remained as significant risk factors for AI (Table 2). The risk of AI was threefold among women with previous OASIS compared to women without (24.4% and 8.1%, resp.).

The higher risk of AI associated with previous OASIS remained threefold in the more severe forms of AI, if defining AI as self-reported St. Mark's score of 5 or above (12.2% and 3.8%) or if 7 or above (7.3% and 2.3%) instead of 3 or above (Table 3).

### 3.3. Women with Previous Cesarean Only

The subgroup of parous women with previous cesarean only (*n* = 140) and no vaginal deliveries was also analyzed separately. In this subgroup the prevalence of AI was the lowest (6.4%, 9/140) compared with all other parity groups but was too small to further analysis of risk factors. When the analyses were repeated with this subgroup of women added to the subgroup of nulliparous women, the conclusions remained unaltered (data not shown).

### 3.4. Women with Dermatological Disease

Women who reported having a dermatological disease reported also more anal incontinence than women without this disease (Table 2). There was a significant association between dermatological disease and several other complaints: allergy, migraine and headache, constipation, and psychological problems. Women who reported dermatological problems also more frequently reported use of vitamins, allergy medication, and stomach and bowel regulators. These women also reported more worries about the Cambridge worry scale than the women without dermatological problems.

## 4. Discussion

This population-based study showed that previous obstetric anal sphincter injury was the strongest risk factor for self-reported AI among pregnant parous women. Among the nulliparous women, a low educational level and comorbidity were associated with anal incontinence. The group of women with previous deliveries with cesarean section only had the lowest prevalence of AI, indicating that pregnancy per se may not represent a major risk factor for AI. These findings support the notion that the process of vaginal delivery may be more damaging to the anal continence mechanisms than pregnancy per se.

We found a surprisingly high frequency of self-reported AI among nulliparous women (7.8%). Low socioeconomic status (low income, low education) is a well-known reason for lower health status and increased morbidity, and previous studies show that self-rated health predicts morbidity well [21–23]; therefore, there is now a reason to doubt the correctness of the self-reported AI. Socioeconomic differences have been found in occurrence of almost all conditions and illnesses [22, 23]. This might explain part of the results for the group of nulliparous pregnant women in our study, where low educational level remained as significant risk factor for AI in the multivariate analysis.

A previous OASIS was the strongest risk factor for self-reported anal incontinence in all analyses in parous women, with and without adjusting for other factors, and in all categories of severity of anal incontinence. In our study, women with previous OASIS reported a lower prevalence of AI than women in previous studies on nonpregnant women [6–8, 24]. All the participants in our study were pregnant, and the low prevalence of AI among women with previous OASIS might indicate that fewer women with severe complaints of AI embark on a new pregnancy [25]. The risk of AI increased with increasing number of vaginal deliveries, a result similar to previous studies. Interestingly, previous delivery with a macrosomic infant (>4000 g) was not associated with self-reported AI during pregnancy in our study, and was not a previous delivery with vacuum extraction or forceps. This is in contrast to some previous studies, where previous forceps delivery and macrosomy are reported as risk factors for AI [8, 26, 27]. In our study of pregnant women, maternal age was not a significant risk factor for AI in the subgroup of parous women, probably because our study group was young, the oldest participant was 45 years old, and age related increased risk of anal incontinence is probably more important in older age groups [2, 5, 27]. Women with overweight (BMI 25–29.9) and obesity (BMI > 30) were more likely to suffer from anal incontinence than women with normal BMI (<25) in our study, but in the multivariate analysis this effect disappeared, due to the strong effect of previous OASIS. The large effect of OASIS exceeded all other factors (except dermatological illness). 

Many previous studies of AI describe only the frequency of the different components of AI. We chose to describe the prevalence of AI as a score, to be able to perform multivariable analyses of the assessed variables in our study. The reason to choose the St. Mark's score 3 as cutoff for AI was to be able to compare the results from this study to our previous study [6] and also to our future study, a followup of the participating women after delivery. As a limit of 3 for defining AI may be questioned for clinical relevance, we repeated all statistical analyses with different cutoffs (4, 5, and 7) for St. Mark's score, for all parity groups. The main conclusions remained unaltered, OASIS was the most important predictor for AI (for all these cutoffs for St. Mark's score) among parous women, and low socioeconomic status and comorbidity were the most important indicators for AI among nulliparous women. Variables with a *P* value over 0.05 were also included in the primary analyses to ensure that no risk factors were missed among our registered variables, but no such factors were revealed.

Similarly to our previous study of nonpregnant women, urinary incontinence was reported by 19.2% of the participants [6]. Prevalence of urinary incontinence was threefold among women who reported AI compared to women without AI, in both nulliparous and parous subgroups of women (*P* < 0.001). This might indicate that some women are in higher overall risk for incontinence, possibly associated with tissue type, or the pathophysiological mechanism may be the same for both diseases.

### 4.1. Strengths and Weaknesses of the Study

Strength of this study is that the pregnant population was unselected and consisted of all parity groups, including nulliparous women. The majority of studies on female anal incontinence have assessed nonpregnant women 6–24 months after delivery. 

Another strength of this study is that we also assessed comorbidity and medication use in addition to obstetrical history. To our knowledge, no previous studies have assessed anal incontinence and comorbidity among pregnant women. Among nulliparous women, comorbidity seems to have association to anal incontinence. Further research is needed to explore whether this is a consistent finding across population groups and to explore which mechanisms could underlie such an association.

A weakness in our study is that the response rate among the invited women was less than 50%, and this can cause self-selection bias among the study participants. Similar selection bias was observed in the Norwegian MoBa study, where higher educated women more likely agreed to participate [28]. However, the effect of such selection bias was found low in the MoBa study [29], and we have no reason to believe that our response rate of 45% negatively affected our study either. Low prevalence of comorbidity and medication use may indicate that the participants did not have lower health status than nonparticipants or the general population in Oslo. 

Bias of women with a previous OASIS and complaints of AI having been more eager to participate in the study is unlikely, since the prevalence of AI in the subgroup of women with previous OASIS was lower (24.4%) than that in the previously reported studies (from Norway) [6, 7]. We compared the study population's basic clinical data with an anonymous electronic database covering all patients delivering at the same time period as the participants in this study. The distribution of nulliparous women in our study population was higher than in the overall delivery population in our hospital (63% and 52%, resp.). We did not find any differences in mean age between responders and nonresponders, but the distribution of women with non-Western background was higher among the nonresponders (data not shown), as expected, as the questionnaire and patient information were in Norwegian.

All data in this study was based on self-reporting from the participants, and thus information of their obstetric history can include errors. It is likely that women remember correctly the number of previous deliveries, delivery mode, and infant birth weight, but not all women are aware of having suffered from OASIS [7] when they leave the hospital after delivery. Lacking information of OASIS might strengthen our conclusions of OASIS being a strong risk factor for AI: if women unaware of previous OASIS reported AI and were analyzed as having no previous OASIS, the risk of AI after OASIS is even higher than calculated in this study. On the other hand, if more women who were unaware of having OASIS reported no AI, our results would show too strong effect of OASIS as a risk factor to AI.

We found an association between self-reported dermatological disease and self-reported AI for all parity groups, which has not been reported before. We can only speculate reasons for this association; women that have AI may also be more sensitive to dermatological bother than others, perhaps associated with the fecal incontinence with affection of perianal skin. Possibly, there could be a common tissue specific risk for both AI and dermatological conditions. Women who reported dermatological problems also reported more worries; another explanation could be that these women were in general more sensitive to different symptoms and signs. In a further study more detailed questions about the type of dermatological disease would be of interest when assessing comorbidity in relation to symptoms of AI.

We have performed a large number of analyses due to a large number of detailed information about obstetric history and maternal characteristics. The study population is relatively young, and thus, frequency of comorbidity and medication use was very low among the participants, which can give results by chance. Therefore, all analyses were also performed without comorbidity and medication use. This did not alter the conclusions; low educational level among nulliparous and OASIS among parous women were the most important factors associated to AI.

We conclude that among parous women, previous OASIS is the most important risk factor for anal incontinence and other obstetrical events only had a minor effect on development of AI. As OASIS is a modifiable risk factor, and frequency may rapidly be altered after introduction of obstetrical perineal support programs [30–33], prevention of obstetrical sphincter injury is likely the most important factor for reducing bothersome anal incontinence in fertile women. Efforts to reduce incidence of OASIS should be highly prioritized in all delivery units.

## Figures and Tables

**Table 1:** Anal incontinence defined in St. Mark's score in subgroups of women. Values are given in frequencies (numbers) or mean/median.

	Nulliparous (n = 1792)		Parous (n = 914)	
	St. Mark's 0–2	St. Mark's 3–16	St. Mark's 0–2	St. Mark's 3–16
Number of women	1653	139	824	90
Born in	*P* < 0.001		*P* < 0.001	
Western country (Western Europe, North America, Oceania)	93.2 (*n* = 1448)	6.8 (*n* = 106)	91.6 (*n* = 717)	8.4 (*n* = 66)
Non-Western country (Asia, Africa, Eastern-Europe, Turkey, or South or Central America)	86.1 (*n* = 198)	13.9 (*n* = 32)	81.7 (*n* = 107)	18.3 (*n* = 24)
Household income, USD	*P* = 0.04		*P* = 0.002	
91,000 or more	93.2 (*n* = 1257)	6.8 (*n* = 91)	92.0 (*n* = 698)	8.0 (*n* = 61)
Less than 91,000	90.0 (*n* = 316)	10.0 (*n* = 35)	82.9 (*n* = 97)	17.1 (*n* = 20)
Marital status	*P* = 0.004		*P* = 0.02	
Married	92.8 (*n* = 632)	7.2 (*n* = 49)	89.7 (*n* = 503)	10.3 (*n* = 58)
Cohabitating	92.6 (*n* = 945)	7.4 (*n* = 76)	93.0 (*n* = 305)	7.0 (*n* = 23)
Unmarried/living alone/single	82.5 (*n* = 66)	17.5 (*n* = 14)	75 (*n* = 15)	25 (*n* = 5)
Maternal educational level	*P* < 0.001		*P* = 0.008	
University 5 years or more	93.1 (*n* = 707)	7.7 (*n* = 136)	93.2 (*n* = 398)	6.8 (*n* = 29)
College/University 4 years	93.2 (*n* = 685)	6.8 (*n* = 50)	90.5 (*n* = 294)	9.5 (*n* = 31)
High school	91.3 (*n* = 219)	8.8 (*n* = 21)	84.5 (*n* = 98)	15.5 (*n* = 18)
Elementary/secondary school	69.8 (*n* = 30)	30.2 (*n* = 13)	81.3 (*n* = 26)	18.8 (*n* = 6)
Working	*P* = 0.57		*P* = 0.09	
Full-time, more than 90%	92.5 (*n* = 1301)	7.5 (*n* = 106)	91.3 (*n* = 619)	8.7 (*n* = 59)
Part-time 40–90%, sick-leave, studying, housewife	93.4 (*n* = 297)	6.6 (*n* = 21)	87.3 (*n* = 186)	12.7 (*n* = 27)
Maternal age mean/median (years)	30.1/30.0	30.6/30.0	33.0/33.0	32.9/34.0
Maternal age	*P* = 0.04		*P* = 0.82	
Less than 35 years	92.8 (*n* = 1427)	7.2 (*n* = 111)	90.3 (*n* = 523)	9.7 (*n* = 56)
35 or more years	89 (*n* = 226)	11.0 (*n* = 28)	89.9 (*n* = 301)	10.1 (*n* = 34)
BMI, mean	23.6	23.8	23.6	24.7
BMI	*P* = 0.64		*P* = 0.03	
16–24.9	92.4 (*n* = 1201)	7.6 (*n* = 99)	91.7 (*n* = 594)	8.3 (*n* = 54)
25–44.4	91.7 (*n* = 443)	8.3 (*n* = 40)	86.9 (*n* = 226)	13.1 (*n* = 34)
Smoking	*P* = 0.93		*P* = 0.73	
No	92.3 (*n* = 1595)	7.7 (*n* = 133)	90.1 (*n* = 810)	9.9 (*n* = 89)
Yes	92.7 (*n* = 38)	7.3 (*n* = 3)	92.9 (*n* = 13)	7.1 (*n* = 1)
Pregnancy duration when answered	*P* = 0.04		*P* = 0.13	
First trimester (6–12 weeks)	88.4 (*n* = 175)	11.6 (*n* = 23)	93.9 (*n* = 124)	6.1 (*n* = 8)
Second or third trimester (13–38)	92.5 (*n* = 1437)	7.5 (*n* = 116)	89.7 (*n* = 672)	10.3 (*n* = 77)
Obstetrical history				
OASIS			*P* = 0.001	
No			90.8 (*n* = 793)	9.2 (*n* = 80)
Yes			75.6 (*n* = 31)	24.4 (*n* = 10)

**Table 2:** Risk of anal incontinence defined in St. Mark's score 3–16 compared to women with St. Mark's score 0–2. Crude OR and adjusted OR with confidence intervals. Adjusted OR is presented for significant variables only.

	Nulliparous (*n* = 1792)		Parous (*n* = 914)	
	Crude OR	Adj. OR (95% CI)	Crude OR	Adj. OR (95% CI)
Born in/maternal origin				
Western (Western Europe, North America, Oseania)	1	1	1	1
Non-Western (Asia, Africa, Eastern-Europe, Turkey, or South or Central America)	2.21 (1.45–3.37)	1.54 (0.85–2.78)	2.44 (1.46–4.06)	1.04 (0.44–2.46)
Household income, NOK/USD				
500 000 or more 91,000	1	1	1	1
Less than 500,000	1.53 (1.02–2.30)	1.07 (0.63–1.80)	2.36 (1.36–4.08)	1.40 (0.63–3.14)
Marital status				
Married	1	1	1	1
Co-habitating	1.04 (0.71–1.51)	1.16 (0.76–1.77)	0.65 (0.39–1.08)	0.78 (0.46–1.36)
Unmarried/living alone/single	2.74 (1.43–5.22)	2.08 (0.91–4.74)	2.89 (1.01–8.24)	2.06 (0.54–7.89)
Maternal educational level				
University 5 years or more	1	1	1	1
College/University 4 years	0.99 (0.66–1.48)	0.91 (0.60–1.40)	1.45 (0.85–2.45)	1.42 (0.81–2.49)
High school	1.30 (0.77–2.21)	1.16 (0.65–2.09)	2.52 (1.34–4.72)	1.85 (0.86–3.99)
Elementary/secondary school	5.89 (2.90–11.97)	3.88 (1.46–10.32)	3.17 (1.21–8.31)	1.00 (0.21–4.03)
Maternal age				
Less than 35 years	1	1	1	
35 or more years	1.59 (1.03–2.47)	1.62 (1.01–2.62)	1.06 (0.67–1.65)	
BMI				
16–24.9	1		1	1
25–44.4	1.10 (0.75–1.61)		1.66 (1.05–2.61)	1.36 (0.81–2.29)
Pregnancy duration when answered				
First trimester (6–12 weeks)	1	1	1	
Second or third trimester (13–38)	0.61 (0.38–0.99)	0.53 (0.32–0.90)	1.78 (0.84–3.77)	
Illness/disease				
Dermatological disease				
No	1	1	1	1
Yes	1.97 (1.15–3.40)	2.39 (1.36–4.20)	2.85 (1.51–5.40)	3.02 (1.51–6.02)
Ulcerative stomach				
No	1	1		
Yes	2.60 (1.13–6.00)	2.42 (0.95–6.15)		
Hypertension				
No	1	1	1	
Yes	2.60 (1.13–6.00)	2.05 (0.77–5.48)	0	
Rheumatoid arthritis or other muscular-skeletal problems				
No	1	1	1	
Yes	2.77 (1.36–5.62)	2.45 (1.14–5.31)	1.83 (0.74–4.51)	
Kidney/urinary problems				
No	1		1	
Yes	1.36 (0.85–2.18)		1.87 (0.94–3.72)	
Medication				
Pain killers				
No	1		1	1
Yes	1–10 (0.50–2.43)		2.40 (1.02–5.66)	1.80 (0.62–5.20)
Obstetrical history				
OASIS				
No			1	1
Yes			3.20 (1.51–6.76)	3.83 (1.68–8.73)
Previous macrosomy, >4000 g				
No			1	
Yes			0.80 (0.42–1.51)	
Previous vacuum extraction				
No			1	
Yes			0.58 (0.29–1.15)	
Forceps				
No			1	
Yes			0.83 (0.19–3.58)	

**Table 3:** Distribution of St. Mark's score among parous women with OASIS and without OASIS.

	Parous women without previous OASIS	Parous women with previous OASIS
	*n* = 873	*n* = 41
St. Mark's 0	81.4 (*n* = 711)	63.4 (*n* = 26)
St. Mark's 1-2	10.4 (*n* = 91)	12.2 (*n* = 5)
St. Mark's 3–16	8.1 (*n* = 71)	24.4 (*n* = 10)
St. Mark's 5–16	3.8 (*n* = 33)	12.2 (*n* = 5)
St. Mark's 7–16	2.3 (*n* = 20)	7.3 (*n* = 3)

## References

[1] Haylen BT, De Ridder D, Freeman RM (2010). An International Urogynecological Association (IUGA)/International Continence Society (ICS) joint report on the terminology for female pelvic floor dysfunction. *International Urogynecology Journal and Pelvic Floor Dysfunction*.

[2] Rommen K, Schei B, Rydning A, Sultan A H, Mørkved S (2012). Prevalence of anal incontinence among Norwegian women: a cross-sectional study. *BMJ Open*.

[3] Memon H, Handa VL (2012). Pelvic floor disorders following vaginal or cesarean delivery. *Current Opinion in Obstetrics and Gynecology*.

[4] Walter S, Hallböök O, Gotthard R, Bergmark M, Sjödahl R (2002). A population-based study on bowel habits in a Swedish community: prevalence of faecal incontinence and constipation. *Scandinavian Journal of Gastroenterology*.

[5] Boreham MK, Richter HE, Kenton KS (2005). Anal incontinence in women presenting for gynecologic care: prevalence, risk factors, and impact upon quality of life. *American Journal of Obstetrics and Gynecology*.

[6] Laine K, Skjeldestad FE, Sanda B, Horne H, Spydslaug A, Staff AC (2011). Prevalence and risk factors for anal incontinence after obstetric anal sphincter rupture. *Acta Obstetricia et Gynecologica Scandinavica*.

[7] Norderval S, Nsubuga D, Bjelke C, Frasunek J, Myklebust I, Vonen B (2004). Anal incontinence after obstetric sphincter tears: incidence in a Norwegian county. *Acta Obstetricia et Gynecologica Scandinavica*.

[8] Huebner M, Gramlich NK, Rothmund R, Nappi L, Abele H, Becker S (2013). Fecal incontinence after obstetric anal sphincter injuries. *International Journal of Gynecology & Obstetrics*.

[9] Faltin DL, Sangalli MR, Curtin F, Morabia A, Weil A (2001). Prevalence of anal incontinence and other anorectal symptoms in women. *International Urogynecology Journal and Pelvic Floor Dysfunction*.

[10] Nelson R, Norton N, Cautley E, Furner S (1995). Community-based prevalence of anal incontinence. *Journal of the American Medical Association*.

[11] Wegnelius G, Hammarström M (2011). Complete rupture of anal sphincter in primiparas: long-term effects and subsequent delivery. *Acta Obstetricia et Gynecologica Scandinavica*.

[12] Hojberg KE, Salvig JD, Winslow NA, Bek KM, Laurberg S, Secher NJ (2000). Flatus and faecal incontinence: prevalence and risk factors at 16 weeks of gestation. *British Journal of Obstetrics and Gynaecology*.

[13] Guise JM, Morris C, Osterweil P, Li H, Rosenberg D, Greenlick M (2007). Incidence of fecal incontinence after childbirth. *Obstetrics and Gynecology*.

[14] Kepenekci I, Keskinkilic B, Akinsu F (2011). Prevalence of pelvic floor disorders in the female population and the impact of age, mode of delivery, and parity. *Diseases of the Colon and Rectum*.

[15] Due U, Ottesen M (2009). The Danish anal sphincter rupture questionnaire: validity and reliability. *Acta Obstetricia et Gynecologica Scandinavica*.

[16] Vaizey CJ, Carapeti E, Cahill JA, Kamm MA (1999). Prospective comparison of faecal incontinence grading systems. *Gut*.

[17] Kulseng-Hanssen S, Borstad E (2003). The development of a questionnaire to measure the severity of symptoms and the quality of life before and after surgery for stress incontinence. *BJOG*.

[18] Hannestad YS, Rortveit G, Sandvik H, Hunskaar S (2000). A community-based epidemiological survey of female urinary incontinence: the Norwegian EPINCONT study. Epidemiology of Incontinence in the County of Nord-Trondelag. *Journal of Clinical Epidemiology*.

[19] Öhman SG, Grunewald C, Waldenström U (2003). Women’s worries during pregnancy: testing the Cambridge Worry Scale on 200 Swedish women. *Scandinavian Journal of Caring Sciences*.

[20] Green JM, Kafetsios K, Statham HE, Snowdon CM (2003). Factor structure, validity and reliability of the Cambridge Worry Scale in a pregnant population. *Journal of Health Psychology*.

[21] Braveman PA, Cubbin C, Egerter S, Williams DR, Pamuk E (2010). Socioeconomic disparities in health in the united States: what the patterns tell us. *American Journal of Public Health*.

[22] Adler NE, Boyce T, Chesney MA (1994). Socioeconomic status and health: the challenge of the gradient. *American Psychologist*.

[23] Van Lenthe FJ, Schrijvers CTM, Droomers M, Joung IMA, Louwman MJ, Mackenbach JP (2004). Investigating explanations of socio-economic inequalities in health: the Dutch GLOBE study. *European Journal of Public Health*.

[24] Roos AM, Thakar R, Sultan AH (2010). Outcome of primary repair of obstetric anal sphincter injuries (OASIS): does the grade of tear matter?. *Ultrasound in Obstetrics and Gynecology*.

[25] Kumar R, Ooi C, Nicoll A (2012). Anal incontinence and quality of life following obstetric anal sphincter injury. *Archives of Gynecology and Obstetrics*.

[26] Pretlove SJ, Thompson PJ, Toozs-Hobson PM, Radley S, Khan KS (2008). Does the mode of delivery predispose women to anal incontinence in the first year postpartum? A comparative systematic review. *BJOG*.

[27] Eogan M, O’Brien C, Daly L, Behan M, O’Connell PR, O’Herlihy C (2011). The dual influences of age and obstetric history on fecal continence in parous women. *International Journal of Gynecology and Obstetrics*.

[28] Magnus P, Irgens LM, Haug K (2006). Cohort profile: the Norwegian mother and child cohort study (MoBa). *International Journal of Epidemiology*.

[29] Nilsen RM, Vollset SE, Gjessing HK (2009). Self-selection and bias in a large prospective pregnancy cohort in Norway. *Paediatric and Perinatal Epidemiology*.

[30] Laine K, Skjeldestad FE, Sandvik L, Staff AC (2012). Incidence of obstetric anal sphincter injuries after training to protect the perineum: cohort study. *BMJ Open*.

[31] Hals E, Øian P, Pirhonen T (2010). A multicenter interventional program to reduce the incidence of anal sphincter tears. *Obstetrics and Gynecology*.

[32] Laine K, Pirhonen T, Rolland R, Pirhonen J (2008). Decreasing the incidence of anal sphincter tears during delivery. *Obstetrics and Gynecology*.

[33] Laine K, Rotvold W, Staff AC (2013). Are obstetric anal sphincter ruptures preventable? Large and consistent rupture rate variations between the Nordic countries and between delivery units in Norway. *Acta Obstetricia et Gynecologica Scandinavica*.

